# Adjustment of Acetabular Cup Inclination Assisted by Smartphone during Revision Total Hip Arthroplasty: Surgical Technique and Report of Four Cases

**DOI:** 10.1055/s-0045-1804490

**Published:** 2025-04-15

**Authors:** Ahmed A. Khalifa, Mahmoud Faisal Adam, Mohamed A. Mahran

**Affiliations:** 1 Departamento de Ortopedia, Qena Faculty of Medicine and University Hospital, South Valley University, Qena, Egito; 2 Departamento de Cirurgia Ortopédica e Traumatologia, Faculty of Medicine, Assiut University, Assiut, Egito; 3 Faculdade de Medicina, Luxor University, New Tiba City, Egito

**Keywords:** acetabulum, arthroplasty, replacement, hip, smartphone

## Abstract

We herein present a smartphone-assisted technique for acetabular cup placement during revision total hip arthroplasty (rTHA). Four patients were operated on: three were submitted to second-stage rTHA after infection, and one underwent surgery due to aseptic loosening. The technique entails three main steps: evaluation of the amount of lateral pelvic tilt (either clinically or radiographically); setting a supracetabular rod using the smartphone app as a reference for inclination adjustment; and acetabulum preparation and final cup placement using the smartphone to guide the inclination angle after considering the amount of pelvic tilt. Cup anteversion was adjusted according to the transverse acetabulum ligament. All 4 cases underwent a follow-up that ranged from 17 to 24 months. None of the patients required further revision, and no complications (intraoperative, early, or late postoperative) were observed. All cups were within the Lewinnek safe zone for inclination (42°, 43°, 47°, and 41°). The functional outcome per the Harris Hip Score was excellent for all patients. Smartphones are cheap tools that can assist physicians in the adjustment of acetabular cup inclination during rTHA; however, assessing the possible lateral pelvic tilt and considering it while placing the cup are crucial.

## Introduction

Proper implant positioning during primary total hip arthroplasty (THA) is one crucial factor for short- and long-term outcomes and survival; 1 2 this becomes more demanding during revision THA (rTHA), especially if there are bone defects or distorted anatomical landmarks. 2 3

The economic burden of rTHA is increasing, and instability and aseptic loosening are the leading causes of revision; therefore, various strategies have been employed to reduce the risk of revision, including improving implant designs, bearing materials, and newer technologies such as computer navigation and robotic-assisted surgeries; however, most of these are expensive and unavailable at every institution. 1 4 5

Smartphone-assisted acetabular cup placement has been investigated in primary THA with promising results both in cadaveric and clinical settings, which showed acceptable accuracy, ease of sue by young surgeons, and cheap application, especially in institutions with economic constraints that limit the introduction of the aforementioned newer technologies. 6 7 8

We herein present a simple and economical technique entailing the use of smartphone applications (apps) to adjust acetabular cup inclination in three steps during rTHA and report the early results of the first four cases.

## Surgical Technique and Description of the Cases

Approval was obtained from our local ethical committee (IRB No.:17300762), and written informed consent was obtained from all patients before surgery.

**Fig. 1:**
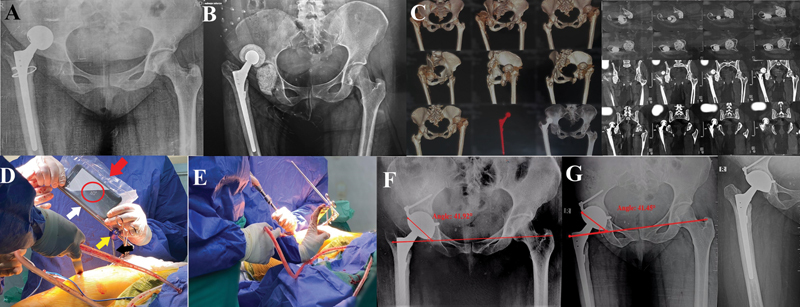
Case 1. ( **A** ) A cementless bipolar hemiarthroplasty with dislocation and infection. ( **B** ) First -stage total hip arthroplasty (THA), with the application of a cement spacer loaded with antibiotics (the stem was retained, as it was solidly ingrown). ( **C** ) Computed tomography (CT) scan to assess the amount of bone defect. ( **D** ) Intraoperative smartphone app-assisted adjustment of the supracetabular rod (white arrow) as a reference for cup inclination (red arrow: smartphone in a sterile plastic bag; red circle: the adjusted angle measured by the Spirit Level app; black arrow: the supraacetabular Schanz screw; and yellow arrow: a clamp). ( **E** ) Acetabular reaming performed parallel to the supraacetabular rod. ( **F** ) Immediate postoperative anteroposterior (AP) radiograph of the pelvis showing cementless dual mobility cup and an inclination angle of 41.9°. ( **G** ) The last follow-up visit (at 24 months), showing maintained cup position.

**Fig. 2:**
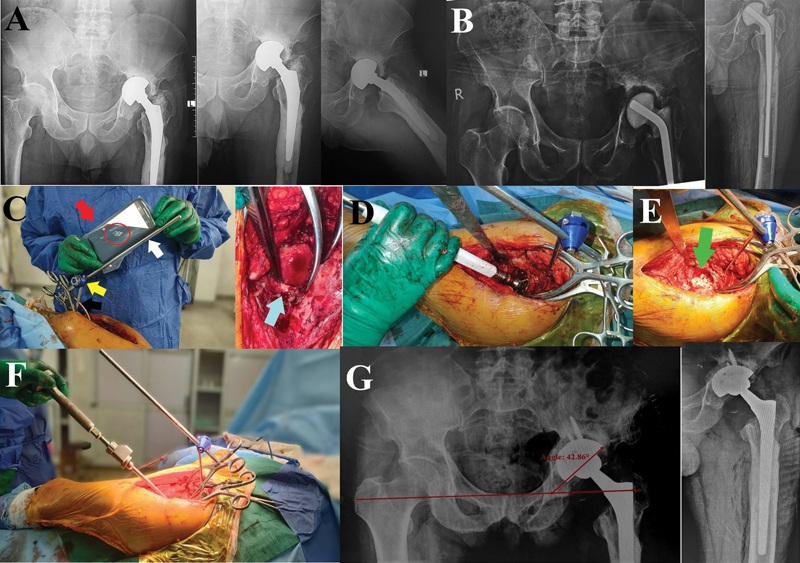
Case 2. ( **A** ) A cemented bipolar hemiarthroplasty with infection after failure of the fixation of a neck of the femur fracture. ( **B** ) First-stage THA with the application of an articulating cement spacer loaded with antibiotics. ( **C** ) Intraoperative smartphone app-assisted adjustment of the supracetabular rod (white arrow) as a reference for cup inclination (red arrow: smartphone in a sterile plastic bag; red circle: the adjusted angle measured by the Spirit Level app; black arrow: the supraacetabular Schanz screw; yellow arrow: a clamp; and blue arrow: transverse acetabular ligament for anteversion adjustment). ( **D** ) Acetabular reaming performed parallel to the supraacetabular rod. ( **E** ) Green arrow showing the allografts used for impaction bone grafting to reconstruct the bone defect. ( **F** ) Final acetabular cup insertion performed while the handle is parallel to the supracetabular rod. ( **G** ) immediate postoperative AP radiographs of the pelvis showing cementless cup and an inclination angle of 42.9°.

**Fig. 3:**
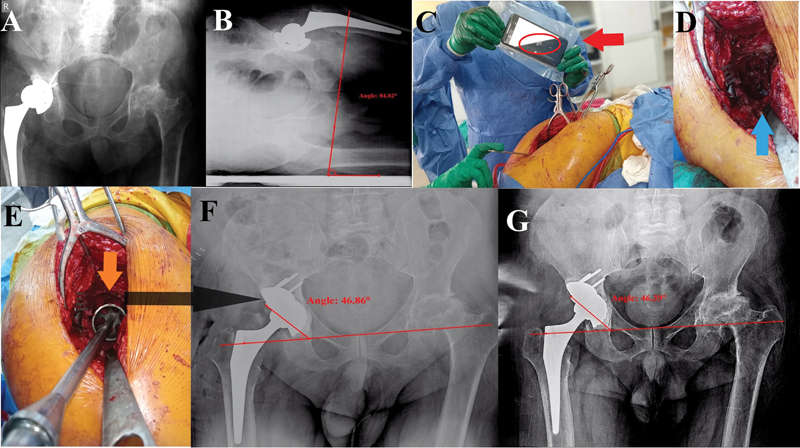
Case 3. ( **A** ) A malpositioned cementless acetabular cup with aseptic loosening. ( **B** ) An AP radiograph of the pelvis with the patient in the lateral decubitus position showing a lateral pelvic tilt (adduction) of -6°. ( **C** ) Intraoperative smartphone app-assisted adjustment of the supracetabular rod as a reference for cup inclination (red arrow: smartphone in a sterile plastic bag; red circle: the adjusted angle measured by the Spirit Level app). ( **D** ) Blue arrow showing the transverse acetabular ligament for anteversion adjustment. ( **E** ) After acetabular reaming and insertion of a trial cup, the amount of acetabular bone defect could be assessed (orange arrow). ( **F** ) Immediate postoperative AP radiograph of the pelvis view showing cemented dual mobility cup with An inclination angle of 46.9°, and bone defect reconstruction using a metal augment. ( **G** ) The last follow-up visit (at 17 months), showing maintained implant position.

**Fig. 4:**
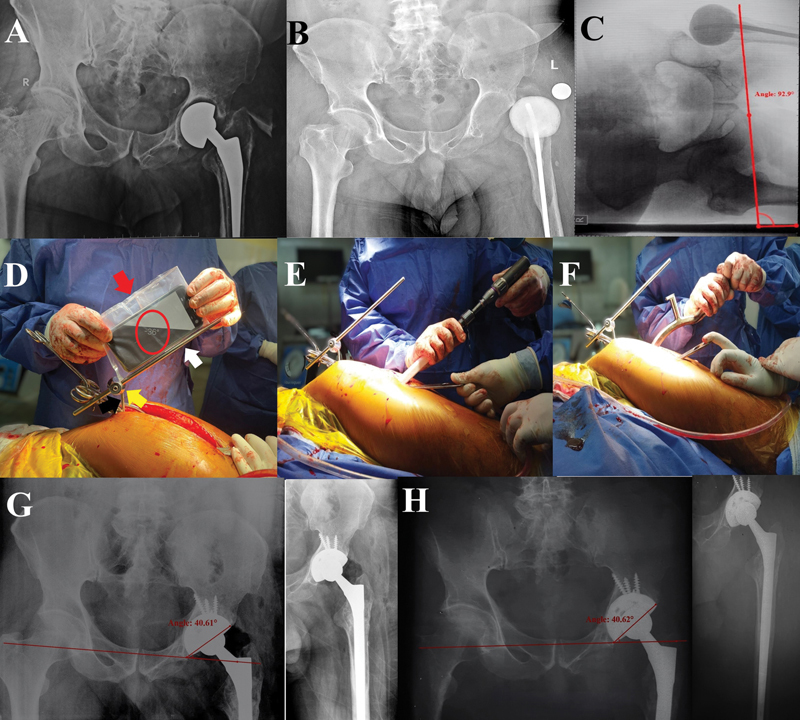
Case 4. ( **A** ) A cemented bipolar hemiarthroplasty with loosening and infection. ( **B** ) First-stage THA with a dislocated cement spacer loaded with antibiotics. ( **C** ) An AP radiograph of the pelvis with the patient in the lateral decubitus position showing a lateral pelvic tilt (abduction) of 3°. ( **D** ) Intraoperative smartphone app-assisted adjustment of the supracetabular rod (white arrow) as a reference for cup inclination (red arrow: smartphone in a sterile plastic bag; red circle: the adjusted angle measured by the Spirit Level app; black arrow: the supraacetabular Schanz screw; and yellow arrow: a clamp). ( **E,F** ) Acetabular reaming and final cup insertion performed parallel to the supraacetabular rod. ( **G** ) Immediate postoperative AP radiograph of the pelvis showing cementless cup and an inclination angle of 40.6°. (H) The last follow-up visit (at 19 months), showing maintained cup position.

The erioperative protocol for all patients was as follows: 1) detailed medical and surgical history, 2) preoperative clinical evaluation; 3) laboratory investigations as part of the regular preoperative assessment and to exclude infection (complete blood count [CBC] with differential white blood cell [WBC] count, erythrocyte sedimentation rate [ESR], and levels of C-reactive protein [CRP]); and 4) imaging studies: plain preoperative radiographs of the pelvis in anteroposterior (AP) view (including both hips; Figs. 1A,B and 2A,B ), of the affected hip in AP and lateral views ( Fig. 2A ), and of the pelvis in AP view while the patient was in the lateral decubitus position (as described in the literature; 9 10 Figs. 3B and 4C ); and computer tomography (CT) scans to assess the amount of bone defect, if suspected ( Fig. 1C ).

The same surgeon operated on all cases (patient details are described in Table 1 and in Figs. 1 2 3 4 ) under spinal anesthesia, with the patient in the lateral decubitus position (after ensuring that the table was parallel to the floor) through a modified direct lateral approach (incorporating the previous surgical incision); after adequate exposure, implant removal, and debridement, at least five tissue samples were obtained and sent for bacterial culture and sensitivity assessment. We aimed at inserting the cup within the Lewinnek safe zone (40° ± 10° for inclination and 15° ± 10° for anteversion).

**Table 1:** Demographic, surgical, and outcome data of the study sample

	Case 1 ( Fig. 1 )	Case 2 ( Fig. 2 )	Case 3 ( Fig. 3 )	Case 4 ( Fig. 4 )
Age (years)	35	61	58	60
Gender	Female	Male	Male	Male
Side	Right	Left	Right	Left
Preoperative diagnosis	PJI requiring second-stage rTHA	PJI requiring second-stage rTHA	Malpositioned cup with aseptic loosening	PJI requiring second-stage rTHA
Number of previous surgeries	3	4	1	3
Follow-up (months)	24	23	17	19
Acetabular bone defect	Yes	Yes	Yes	Yes
Bone defect per the Paprosky classification	2A	2C	3A	2A
Preoperative lateral pelvic tilt (degrees)	0	0	-6	+3
Implants used	Cementless dual mobility cup, the femoral side was not revised	Cementless primary cup and cementless stem (Wagner)	Cemented dual mobility cup, the femoral side was not revised	Cementless primary cup and cementless stem (Wagner)
Bone defect reconstruction	Not required	Impaction bone grafting using allograft	Metal (tantalum) augment	Not required
Postoperative cup inclination (degrees)	41.9	42.9	46.9	40.6
Cup inclination at the last follow-up (degrees)	41.5	Not acquired	46.4	40.6
Complications	Mild, occasional pain and limping gait	Mild, occasional pain	None	None
Harris Hip Score (at the last follow-up)	95	Not acquired*	92	90

**Abbreviations:** PJI, periprosthetic joint infection; rTHA, revision total hip arthroplasty.

**Note:** *The patient could not come for follow-up; we contacted him by phone, and he reported returning to his daily activities, occasional pain, sporadic use of a cane while walking, but no symptoms suggestive of infection.

The three main steps of smartphone-assisted adjustment of acetabular cup inclination during rTHA are as follows:

1) Radiological or clinical calculation of the possible lateral pelvic tilt; radiological: in the pelvis AP radiograph with the patient in the lateral decubitus position, as the angle between a transverse pelvis axis (interteardrop or interischial lines) and the level of the radiology table ( Figs. 3B and 4C ); if it cannot be obtained preoperatively, it can be obtained using fluoroscopy after final positioning of the patient on the operative table. The pelvis could be in a neutral position (0°) if the angle is of 90°, abducted (positive value) if the angle is > 90°, and adducted (negative value) if the angle is < 90°; 8 9 10 clinical: as an angle between a line connecting marks placed on the anterosuperior iliac spines (ASISs) bilaterally and the level of the operative table. 6 8
2) Setting the intraoperative reference for cup inclination: we followed steps described previously in the literature, 6 8 entailing the use of a sterile plastic bag as a protector for the smartphone after turning on the Spirit Level app with a built-in compass on an iPhone XR smartphone (Apple Inc., Cupertino, CA, United States), or other similar, free, and downloadable apps (for Android platform-based smartphones). A supra-acetabular Schanz screw was inserted from within the surgical approach to which a rod was connected using an adjustable clamp. The rod acts as the reference to adjust the cup inclination after considering the value of the pelvic tilt measured. For a target final inclination angle of 45° and if the pelvic tilt is of -10°, for example, we adjust the rod at 35°, and vice versa for pelvic abduction ( Figs. 1D, 2C, 3C , and 4D ).
3) Acetabulum reaming and final acetabular cup insertion: acetabulum reaming in a progressed manner was performed by adjusting the reamer handle parallel to the supraacetabular rod for inclination adjustment ( Figs. 1E, 2D , and 4E ). For the anteversion, we rely mainly on the transverse acetabular ligament (TAL; Figs. 2C and 3D ), a consistent anatomical landmark in nearly most revision cases. 11 12 After reaching a proper fit, a trial acetabular component is used to assess the final cup size, the stability, and the need to reconstruct the present acetabular defect ( Fig. 3E ). The final cup insertion (cemented or cementless) is performed while the inserter handle is parallel to the supraacetabular rod ( Figs. 2F and 4F ).

### Postoperative Assessment and Follow-up

Postoperatively and at the last follow-up visit, AP radiographs of the pelvis were obtained to evaluate the inclination (abduction) angle of the acetabular cup, measured between the interteardrop or interischial lines and a line along the axis of the cup eclipse formed by the superolateral edge and inferomedial edge as reference points ( Figs. 1F, 2G, 3F , and 4G ). The functional outcomes at the last follow-up visit were assessed according to the Harris Hip Score (HHS), and complications at any point of the follow-up were reported. The outcomes are shown in Table 1 .

## Discussion

Most surgeons agree that optimum acetabular cup positioning is crucial for long-term results and to reduce the incidence of instability after primary and revision THA. 2 5 11

The use of computer navigation and robotics in rTHA showed satisfactory results regarding the decrease in the risk of instability due to proper placement of the implants and the reduction in dislocation rates after rTHA of up to 0%. 13 However, these technologies are expensive, unavailable in every institution, and require specific training and preparation. 4 5

To overcome these obstacles, we have applied smartphone apps that successfully assisted acetabular cup adjustment in four rTHA surgeries. We believe that the technique is simple and surgeon-friendly without the need for complex preparation or special preoperative imaging studies apart from AP radiographs of the pelvis with the patient in the lateral decubitus position to calculate the lateral pelvic tilt, and helped in achieving the acetabular cup inclination angle within the safe zone; furthermore, no complications or infections were reported in any of the cases.

The smartphone-assisted cup placement technique has described in primary THA, and it showed promising results in terms of helping young, less experienced surgeons obtain optimum acetabular cup placement comparable to their senior peers, with further improvement in cup placement accuracy compared with visual methods; this was proven in clinical studies, 8 as well as in an invitro and cadaveric models. 7

In the current technique, we employed the same manual instruments used routinely during THAs, without the need for a complex setup. The time spent using the smartphone app and adjusting the angles was of approximately 5 minutes, without external assistance. We admit that the smartphone-assisted technique cannot compete with the accuracy of computer navigation or robotic-assisted cup placement; however, we believe it is more economical and time-saving than these technologies.

In a systematic review on the role of an inclinometer (including smartphone apps) in the adjustment of acetabular cup positioning during THA, van Duren et al. 3 reported that, in the inclinometer group, the cup inclination angle was significantly more within the target zone compared with the freehand or mechanical guide-assisted techniques. They 3 also reported that using an inclinometer increased the operative time by 2 to 7 minutes compared with other techniques based on the results of 3 clinical studies.

The amount of lateral pelvic tilt was reported to reach ± 10° in the literature, with up to 45% of the patients having an absolute tilt of 5°. 8 9 14 Therefore, one crucial preoperative step we recommend is to anticipate the amount of lateral pelvic tilt, which could pass unnoticed by the surgeon and get obscured after draping, especially in overweight patients or when there is a fixed hip joint deformity. 9 10 14

We obtained acetabular cup inclination within the safe zones in all patients, and no instability was reported during the follow-up. Kurosaka et al. 7 compared the accuracy of iPhone-assisted acetabular cup placement to computer navigation in five cadaveric hips in procedures performed by 7 surgeons (4 first-year residents and 3 senior hip surgeons); they reported a mean difference between both techniques of 2.1° ± 1.6° (range: 0°–6°), no significant difference between residents or senior surgeons in inclination adjustment ( *p* = 0.74), and that all acetabular cups placed using the iPhone technique were within the Lewinnek safe zone. 7

One limitation of the smartphone technique is the difficulty in assessing anteversion, as reported in previous studies. 7 To overcome this obstacle, we relied on the TAL in all cases as a consistent patient-specific anatomical landmark. 12

## Conclusion

Smartphones can assist young surgeons or those who do not have access to newer technologies in the adjustment of acetabular cup inclination when placing the cup in rTHA; however, assessing the possible lateral pelvic tilt and considering it while placing the cup are crucial.
