# Supplementary material for: Adjustment of Acetabular Cup Inclination Assisted by Smartphone during Revision Total Hip Arthroplasty: Surgical Technique and Report of Four Cases
Source: Rev Bras Ortop (Sao Paulo). 2025 Apr 15;60(1):s00451804490. doi: 10.1055/s-0045-1804490 (PMC12020590; doi:10.1055/s-0045-1804490)
Supplement: Related texts

# Research article: Ajuste de inclinação do acetábulo assistido por *smartphone* durante revisão de artroplastia total de quadril: Técnica cirúrgica e relato de quatro casos

**Keywords:** acetábulo, artroplastia do quadril, smartphone

## Abstract

Apresentamos Uma Técnica Assistida Por *Smartphone* Para A Colocação De Cúpula Acetabular Durante A Revisão De Artroplastia Total De Quadril (Ratq). Quatro Pacientes Foram Submetidos À Cirurgia; Três à rATQ de segundo estágio após infecção e um devido a afrouxamento asséptico. A técnica tem três etapas principais: avaliação (clínica ou radiográfica) da inclinação pélvica lateral; colocação de uma haste supra-acetabular usando o aplicativo do *smartphone* como referência para o ajuste da inclinação; e preparação do acetábulo e colocação final da cúpula utilizando o *smartphone* para a orientação do ângulo de inclinação após considerar a inclinação pélvica. A anteversão da cúpula foi ajustada de acordo com o ligamento acetabular transverso. Todos os quatro casos foram acompanhados por 17 a 24 meses; nenhum paciente precisou de outra cirurgia de revisão ou apresentou complicações (intraoperatórias pós-operatórias precoces ou tardias). Todas as cúpulas estavam dentro da zona segura de Lewinnek de inclinação (42° 43° 47° e 41°). O resultado funcional de acordo com o Harris Hip Score foi excelente em todos os pacientes. Os *smartphones* são ferramentas baratas que podem auxiliar os médicos no ajuste da inclinação da cúpula acetabular durante a rATQ; no entanto é crucial avaliar a possível inclinação pélvica lateral e considerá-la ao posicionar a cúpula.

## Introdução

O posicionamento adequado do implante durante a artroplastia total do quadril (ATQ) primária é crucial para os desfechos e a sobrevida no curto e longo prazos. 1 2 É ainda mais importante durante a revisão de ATQ (rATQ), em especial na presença de defeitos ósseos ou distorção dos marcos anatômicos. 2 3

O fardo econômico da rATQ tem aumentado graças à instabilidade e ao afrouxamento asséptico, que são as principais causas de revisão. Portanto, várias estratégias já foram empregadas para reduzir o risco de revisão, inclusive a melhora do desenho dos implantes, dos materiais de suporte e novas tecnologias, como navegação por computador e cirurgias assistidas por robótica. No entanto, a maioria dessas tecnologias é cara e não está disponível em todas as instituições. 1 4 5

A colocação de acetábulo assistida por *smartphone* foi investigada na ATQ primária com resultados promissores tanto em estudos com cadáveres quanto com pacientes, e demonstrou precisão aceitável, facilidade de uso por cirurgiões jovens e aplicação barata, particularmente em instituições com algumas restrições econômicas que limitam a introdução das novas tecnologias já mencionadas. 6 7 8

Apresentamos uma técnica simples e econômica com o uso de aplicativos de *smartphone* para o ajuste da inclinação do acetábulo em uma técnica de rATQ de três etapas, e relatamos os resultados obtidos nos quatro primeiros casos.

## Técnica Cirúrgica e Descrição dos Casos

Este estudo foi aprovado pelo comitê de ética institucional (IRB número: 17300762), e o termo de consentimento livre e esclarecido foi assinado por todos os pacientes antes da cirurgia.

O protocolo perioperatório para todos os pacientes foi o seguinte: 1) histórico médico e cirúrgico detalhados; 2) avaliação clínica pré-operatória; 3) exames laboratoriais como parte da avaliação pré-operatória regular e para a exclusão de infecção (na forma de hemograma completo com contagem diferencial de leucócitos, velocidade de hemossedimentação e determinação da concentração de proteína C-reativa); e 4) exames de imagem: radiografias simples pré-operatórias em projeções anteroposterior (AP) da pelve (incluindo ambos os quadris) ( Figs. 1A,B e 2A,B ), AP e em perfil do quadril afetado ( Fig. 2A ), e AP da pelve com o paciente em decúbito lateral (conforme descrito na literatura; 9 10 Figs. 3B e 4C ). Também solicitamos tomografias computadorizadas (TC) para a avaliação da extensão do defeito ósseo em caso de suspeita ( Fig. 1C ).

**Fig. 1:** Caso 1. ( **A** ) Hemiartroplastia bipolar não cimentada com deslocamento e infecção. ( **B** ) Artroplastia total de quadril (ATQ) de primeiro estágio com a aplicação de um espaçador de cimento com antibióticos (a haste foi retida, pois sua posição era sólida). (C) Tomografia computadorizada para a avaliação da extensão do defeito ósseo. (D) Ajuste intraoperatório assistido por aplicativo de *smartphone* da haste supra-acetabular (seta branca) como referência para a inclinação da cúpula (seta vermelha: *smartphone* em saco plástico estéril; círculo vermelho: ângulo ajustado medido pelo aplicativo Spirit Level; seta preta: parafuso de Schanz supra-acetabular; e seta amarela: pinça). ( **E** ) Fresagem acetabular paralela à haste supra-acetabular. ( **F** ) Radiografia em incidência anteroposterior (AP) da pelve no período pós-operatório imediato, mostrando cúpula de mobilidade dupla sem cimento e ângulo de inclinação de 41,9°. ( **G** ) Último acompanhamento (aos 24 meses), mostrando a manutenção da posição da cúpula.

**Fig. 2:** Caso 2. (A) Hemiartroplastia bipolar cimentada com infecção após a fixação de fratura do colo do fêmur prévia e malsucedida. ( **B** ) Artroplastia total de quadril de primeiro estágio com a aplicação de um espaçador de cimento articulado com antibióticos. ( **C** ) Ajuste intraoperatório assistido por aplicativo de *smartphone* da haste supra-acetabular (seta branca) como referência para a inclinação da cúpula (seta vermelha: *smartphone* em um saco plástico estéril; círculo vermelho: ângulo ajustado medido pelo aplicativo Spirit Level; seta preta: parafuso de Schanz supra-acetabular; seta amarela: pinça; e seta azul: ligamento acetabular transverso para o ajuste da anteversão). ( **D** ) Fresagem acetabular paralela à haste supra-acetabular. ( **E** ) Seta verde mostrando os aloenxertos usados para o enxerto ósseo de impacto para a reconstrução do defeito ósseo. ( **F** ) Inserção final da cúpula acetabular com o cabo paralelo à haste supra-acetabular. (G) Radiografia AP da pelve no período pós-operatório imediato, mostrando a cúpula sem cimento e um ângulo de inclinação de 42,9°.

**Fig. 3:** Caso 3. ( **A** ) Cúpula acetabular não cimentada, mal posicionada, e com afrouxamento asséptico. ( **B** ) Radiografia AP da pelve com o paciente em decúbito lateral, mostrando a inclinação pélvica lateral (adução) de -6°. ( **C** ) Ajuste intraoperatório assistido por aplicativo de *smartphone* da haste supra-acetabular como referência para a inclinação da cúpula (seta vermelha: *smartphone* em saco plástico estéril; círculo vermelho: ângulo ajustado medido pelo aplicativo Spirit Level). ( **D** ) Seta azul mostrando o ligamento acetabular transverso para o ajuste da anteversão. ( **E** ) Após a fresagem acetabular e a inserção de uma cúpula de teste, a extensão do defeito ósseo acetabular pôde ser avaliada (seta laranja). ( **F** ) Radiografia AP da pelve no período pós-operatório imediato, mostrando a cúpula de mobilidade dupla cimentada com ângulo de inclinação de 46,9° e reconstrução do defeito ósseo com um aumentador metálico. ( **G** ) Último acompanhamento (aos 17 meses), mostrando a manutenção da posição da cúpula.

**Fig. 4:** Caso 4. ( **A** ) Hemiartroplastia bipolar cimentada com soltura e infecção. ( **B** ) Artroplastia total de quadril de primeiro estágio com um espaçador de cimento deslocado e com antibióticos. ( **C** ) Radiografia AP da pelve com o paciente em decúbito lateral, mostrando a inclinação pélvica lateral (abdução) de 3°. ( **D** ) Ajuste intraoperatório assistido por aplicativo de *smartphone* da haste supra-acetabular (seta branca) como referência para a inclinação da cúpula (seta vermelha: *smartphone* em saco plástico estéril; círculo vermelho: ângulo ajustado medido pelo aplicativo Spirit Level, seta preta: parafuso de Schanz supra-acetabular; e seta amarela: pinça). ( **E,F** ) Fresagem acetabular e inserção final da cúpula paralelas à haste supra-acetabular. ( **G** ) Radiografia AP da pelve no período pós-operatório imediato, mostrando a cúpula sem cimento e ângulo de inclinação de 40,6°. ( **H** ) Último acompanhamento (aos 19 meses), mostrando a manutenção da posição da cúpula.

O mesmo cirurgião foi responsável por todos os procedimentos (os detalhes dos pacientes estão descritos na Tabela 1 e nas Figs. 1 2 3 4 ), realizados sob raquianestesia com o paciente em decúbito lateral (após assegurar o paralelismo da mesa em relação ao chão) por meio de uma abordagem lateral direta modificada (incorporando a incisão cirúrgica anterior); após a exposição adequada, a remoção do implante e o desbridamento, pelo menos cinco amostras de tecido foram obtidas e enviadas para cultura bacteriana e antibiograma. Nosso objetivo era inserir a cúpula dentro da zona de segurança de Lewinnek (40° ± 10° de inclinação e 15° ± 10° de anteversão).

**Tabela 1:** Dados demográficos, cirúrgicos e dos desfechos da amostra do estudo

	Caso 1 ( Fig. 1 )	Caso 2 ( Fig. 2 )	Caso 3 ( Fig. 3 )	Caso 4 ( Fig. 4 )
Idade (anos)	35	61	58	60
Sexo	Feminino	Masculino	Masculino	Masculino
Lado	Direito	Esquerdo	Direito	Esquerdo
Diagnóstico pré-operatório	IAP que demandava rATQ de segundo estágio	IAP que demandava rATQ de segundo estágio	Cúpula mal posicionada com afrouxamento asséptico	IAP que demandava rATQ de segundo estágio
Número de cirurgias anteriores	3	4	1	3
Período de acompanhamento (meses)	24	23	17	19
Defeito no osso acetabular	Sim	Sim	Sim	Sim
Defeito ósseo segundo a classificação de Paprosky	2A	2C	3A	2A
Inclinação pélvica lateral pré-operatória (graus)	0	0	-6	+3
Implantes usados	Cúpula não cimentada de mobilidade dupla; o lado femoral não foi revisto	Cúpula primária cimentada e haste não cimentada (Wagner)	Cúpula cimentada de mobilidade dupla; o lado femoral não foi revisto	Cúpula primária cimentada e haste não cimentada (Wagner)
Reconstrução do defeito ósseo	Não necessária	Enxerto ósseo de impacto com aloenxerto	Aumentador metálico (tântalo)	Não necessária
Inclinação pós-operatória da cúpula (graus)	41,9	42,9	46,9	40,6
Inclinação da cúpula no último acompanhamento (graus)	41,5	Não obtida	46,4	40,6
Complicações	Dor branda ocasional e claudicação	Dor branda ocasional	Nenhuma	Nenhuma
Harris Hip Score (no último acompanhamento)	95	Não obtido*	92	90

**Abreviaturas:** IAP, infecção articular periprotética; rATQ, revisão de artroplastia total do quadril.

**Nota:** *O paciente não pôde comparecer ao acompanhamento; em contato telefônico, o paciente relatou retorno às suas atividades diárias, dor ocasional, uso esporádico de bengala ao caminhar, mas nenhum sintoma sugestivo de infecção.

Os detalhes cirúrgicos, nas três etapas principais, para a utilização de aplicativos de *smartphone* na rATQ para o ajuste da inclinação do acetábulo, foram:

1) Cálculo radiológico ou clínico da possível inclinação lateral da pelve; radiológico: na radiografia AP da pelve, com o paciente em decúbito lateral, como o ângulo entre um eixo transversal da pelve (linhas interlágrimas ou interisquiáticas) e o nível da mesa de radiologia ( Figs. 3B e 4C ); caso não possa ser obtida antes da cirurgia, pode ser obtida por fluoroscopia após o posicionamento final do paciente na mesa cirúrgica. A pelve pode estar em posição neutra (0°) se o ângulo for de 90°, abduzida (valor positivo), se o ângulo for > 90° , ou aduzida (valor negativo), se o ângulo for < 90°; 8 9 10 clínica: como o ângulo entre uma linha que conecta as marcas nas espinhas ilíacas anterossuperiores (EIAS) bilaterais e o nível da mesa cirúgica. 6 8
2) Definição da referência intraoperatória para a inclinação da cúpula: seguimos os mesmos passos descritos na literatura, 6 8 usando um saco plástico estéril para proteger o *smartphone* após o acionamento do aplicativo Spirit Level, com uma bússola embutida em um iPhone XR (Apple Inc., Cupertino, CA, Estados Unidos) ou outros aplicativos similares de *download* gratuito (para *smartphones* baseados na plataforma Android). Um parafuso de Schanz supra-acetabular foi inserido de dentro da abordagem cirúrgica e conectado a uma haste com uma braçadeira ajustável. A haste é uma referência para ajustar a inclinação da cúpula após considerar o valor da inclinação pélvica. Para um ângulo-alvo de inclinação final de 45° e se a inclinação pélvica for de -10°, por exemplo, a haste é ajustada em 35° e vice-versa, em caso de abdução pélvica ( Figs. 1D, 2C, 3C e 4D ).
3) Fresagem do acetábulo e inserção final da cúpula acetabular: a fresagem progressiva do acetábulo foi realizada por meio de ajuste do cabo da fresa paralelamente à haste supra-acetabular para o ajuste da inclinação ( Figs. 1E, 2D e 4E ). Para a anteversão, contamos principalmente com o ligamento acetabular transverso (LAT) ( Figs. 2C e 3D ), um marco anatômico consistente na maioria dos casos de revisão. 11 12 Após atingir o ajuste adequado, um componente acetabular de teste é usado para avaliar o tamanho final da cúpula, a estabilidade, e a necessidade de reconstrução do defeito acetabular presente ( Fig. 3E ). A inserção final da cúpula (cimentada ou não) é realizada com o cabo do equipamento de inserção paralelo à haste supra-acetabular ( Figs. 2F e 4F ).

## Avaliação e Acompanhamento Pós-operatórios

No pós-operatório e no último acompanhamento, radiografias AP da pelve foram obtidas para avaliar o ângulo de inclinação (abdução) da cúpula acetabular, medido entre as linhas interlágrimas ou interisquiáticas e uma linha ao longo do eixo do eclipse da cúpula, formado pela borda superolateral e pela borda inferomedial como pontos de referência ( Figs. 1F, 2G, 3F e 4G ). Os resultados funcionais no último acompanhamento foram avaliados de acordo com o Harris Hip Score (HHS), e complicações em qualquer ponto do acompanhamento foram relatadas. Os desfechos estão apresentados na Tabela 1 .

## Discussão

A maioria dos cirurgiões concorda que o posicionamento ideal do acetábulo é crucial para os desfechos em longo prazo e a redução da incidência de instabilidade após ATQ primária e de revisão. 2 5 11

A navegação por computador e a utilização de robótica na rATQ mostraram resultados satisfatórios em relação à diminuição do risco de instabilidade por posicionamento adequado dos implantes e à redução das taxas de luxação após a rATQ em até 0%. 13 No entanto, essas tecnologias são dispendiosas, não estão disponíveis em todas as instituições ,e exigem formação e preparação específicas. 4 5

Para superar esses obstáculos, usamos aplicativos de *smartphone* que auxiliaram com sucesso o ajuste da cúpula acetabular em quatro procedimentos de rATQ. Acreditamos que a técnica é simples e de fácil aplicação pelo cirurgião, sem a necessidade de preparação complexa ou exames especiais de imagem antes do procedimento além de uma radiografia AP da pelve com o paciente em decúbito lateral para o cálculo da inclinação pélvica lateral, e ajudou a atingir o ângulo de inclinação da cúpula acetabular dentro da zona segura; além disso, nenhuma complicação ou infecção foi relatada em nenhum dos casos.

A técnica de colocação de cúpula assistida por *smartphone* foi descrita na ATQ primária e mostrou resultados promissores ao ajudar cirurgiões jovens e menos experientes a alcançar a colocação ideal da cúpula acetabular de modo comparável ao dos seus pares mais experientes, com melhora na precisão do procedimento em comparação a métodos visuais. Isso foi comprovado em estudos clínicos, 8 *in vitro* e em modelos com cadáveres. 7

Na técnica atual, usamos os mesmos instrumentos manuais das ATQs de rotina, sem a necessidade de configuração complexa. O tempo gasto utilizando o aplicativo do *smartphone* e ajustando os ângulos foi de cerca de 5 minutos, sem assistência externa. Admitimos que a técnica assistida por *smartphone* não pode competir com a precisão da navegação por computador ou o posicionamento da cúpula assistido por robótica; no entanto, acreditamos que é mais econômica e reduz o tempo gasto com essas tecnologias.

Em uma revisão sistemática sobre o papel de um inclinômetro (incluindo aplicativos de *smartphone* ) no ajuste do posicionamento da cúpula acetabular durante a ATQ, van Duren et al. 3 relataram que o grupo em que foi utilizado o inclinômetro apresentou ângulos de inclinação da cúpula significativamente mais próximos da zona-alvo em comparação às técnicas assistidas por guias mecânicos ou à mão livre. Eles 3 também relataram que o uso do inclinômetro aumentou o tempo operatório em 2 a 7 minutos em comparação a outras técnicas, com base nos resultados relatados em 3 ensaios clínicos.

Na literatura, a inclinação pélvica lateral foi relatada como ± 10°, com até 45% dos pacientes apresentando uma inclinação absoluta de 5°. 8 9 14 Portanto, uma etapa pré-operatória crucial que recomendamos é antecipar a inclinação pélvica lateral, que pode passar despercebida pelo cirurgião e ficar obscurecida após a colocação dos curativos, especialmente em pacientes com sobrepeso ou deformidade fixa da articulação do quadril. 9 10 14

Obtivemos inclinações da cúpula acetabular dentro das zonas seguras em todos os pacientes, e nenhuma instabilidade foi relatada durante o acompanhamento. Kurosaka et al. 7 compararam a precisão do posicionamento da cúpula acetabular assistida por iPhone à navegação por computador em cinco quadris de cadáveres em procedimentos realizados por sete cirurgiões (quatro residentes do primeiro ano e três cirurgiões de quadril seniores); eles relataram uma diferença média entre as técnicas de 2,1° ± 1,6° (variação: 0°–6°), mas nenhuma diferença significativa entre residentes ou cirurgiões experientes no ajuste de inclinação ( *p* = 0,74). Além disso, todas as cúpulas acetabulares colocadas usando a técnica com o iPhone estavam dentro da zona segura de Lewinnek. 7

Uma limitação da técnica do *smartphone* é a dificuldade de avaliação da anteversão, já relatada em estudos anteriores. 7 Para superar esse obstáculo, confiamos no LAT em todos os casos como um marco anatômico consistente e específico do paciente. 12

## Conclusão

*Smartphones* podem auxiliar cirurgiões jovens ou que não têm acesso a tecnologias mais recentes no ajuste da inclinação da cúpula acetabular quando de sua colocação na rATQ. No entanto, a avaliação da possível inclinação pélvica lateral e sua consideração durante o posicionamento da cúpula são cruciais.
